# Atomic Resolution Crystal Structure of NAD^+^-Dependent Formate Dehydrogenase from Bacterium *Moraxella sp. C-1*

**Published:** 2009-10

**Authors:** I.G. Shabalin, K.M. Polyakov, V.I. Tishkov, V.O. Popov

**Affiliations:** 1 A.N. Bach Institute of Biochitalicistry RAS;; 2 V.A. Engelhardt Institute of Molecular Biology RAS

## Abstract

The crystal structure of the ternary complex of NAD+-dependent formate dehydrogenase from the methylotrophic bacterium Moraxella sp. C-1 with the cofactor (NAD+) and the inhibitor (azide ion) was established at 1.1 A resolution. The complex mimics the structure of the transition state of the enzymatic reaction. The structure was refined with anisotropic displacitalicents parameters for non-hydrogen atoms to a R factor of 13.4%. Most of the nitrogen, oxygen, and carbon atoms were distinguished based on the analysis of the titalicperature factors and electron density peaks, with the result that side-chain rotamers of histidine residues and most of asparagine and glutamine residues were unambiguously determined. A comparative analysis of the structure of the ternary complex determined at the atomic resolution and the structure of this complex at 1.95 A resolution was performed. In the atomic resolution structure, the covalent bonds in the nicotinamide group are somewhat changed in agreitalicent with the results of quantum mechanical calculations, providing evidence that the cofactor acquires a bipolar form in the transition state of the enzymatic reaction.

## INTRODUCTION

Formate dehydrogenases catalyze the oxidation of formate into carbon dioxide and can be divided into several groups based on the quaternary structure, as well as on the presence of prosthetic groups and cofactors. The structurally simplest formate dehydrogenases are NAD^+^-dependent formate dehydrogenases (FDH, EC 1.2.1.2), which oxidize formate coupled with reduction of the coenzyme NAD^+^ into NADH [1]

HCOO^-^ + NAD^+^ - CO2 ^ + NADH.

Formate dehydrogenases belong to a large superfamily of D-isomer specific 2-hydroxyacid dehydrogenases [2]. Formate dehydrogenases of this type contain no metal ions or prosthetic groups in the active sites and have a high specificity towards both NAD^+^ and formate. FDHs from different organisms (bacteria, yeast, plants) function as dimers consisting of two identical subunits with a molecular weight from 35 to 50 kDa. The molecular mechanism of FDH is characterized by the direct transfer of a hydride ion from the substrate to the C4 atom of the nicotinamide ring of NAD^+^ without additional proton transfer steps, which usually occurs in reactions catalyzed by related NAD^+^-dependent dehydrogenases. Hence, the FDH-catalyzed reaction is a convenient model for studying the mechanism of hydride ion transfer in the active site of NAD^+^-dependent hydrogenases by methods of quantum mechanics and molecular dynamics [3-5].

The knowledge of the three-dimensional structure of the enzyme under study with accurate atomic coordinates is of great importance for the investigation of the molecular mechanisms of the enzyme. Based on X-ray diffraction data at atomic resolution, the protein structure can be refined with anisotropic displacitalicent parameters for individual non-hydrogen atoms, which substantially increases the reliability of structural information and allows the determination of atomic coordinates with 0.02 A accuracy [6]. At this resolution, it is possible to reveal the fine-structural organization of the active site, which cannot be done at lower resolution.

Currently, 937 protein structures with atomic resolution are available in the Protein Data Bank (PDB, http://www.rcsb.org). This is approximately 1.6 % of the total number of structures in the PDB. Atomic resolution structures were solved only for three NAD^+^-dependent dehydrogenases, such as horse liver alcohol dehydrogenase (five structures) [7-9], R-specific alcohol dehydrogenase from *Lactobacillus brevis* (three structures) [10], and lactate dehydrogenase from *Plasmodium falciparum* (one structure) [11]. The available three-dimensional structures of FDH were determined at resolutions no higher than 1.8 A for the holo-form [12] and 1.55 A for the apo-form [13]. In this work, the three-dimensional structure of the ternary complex of formate dehydrogenase from the methylotrophic bacterium *Moraxella sp. C-1* (MorFDH) with NAD^+^ and an inhibitor (azide ion), which mimics the transition state of the enzymatic reaction, was established at atomic resolution (1.1 A).

## MATERIALS AND METHODS

**Fig. 1.**
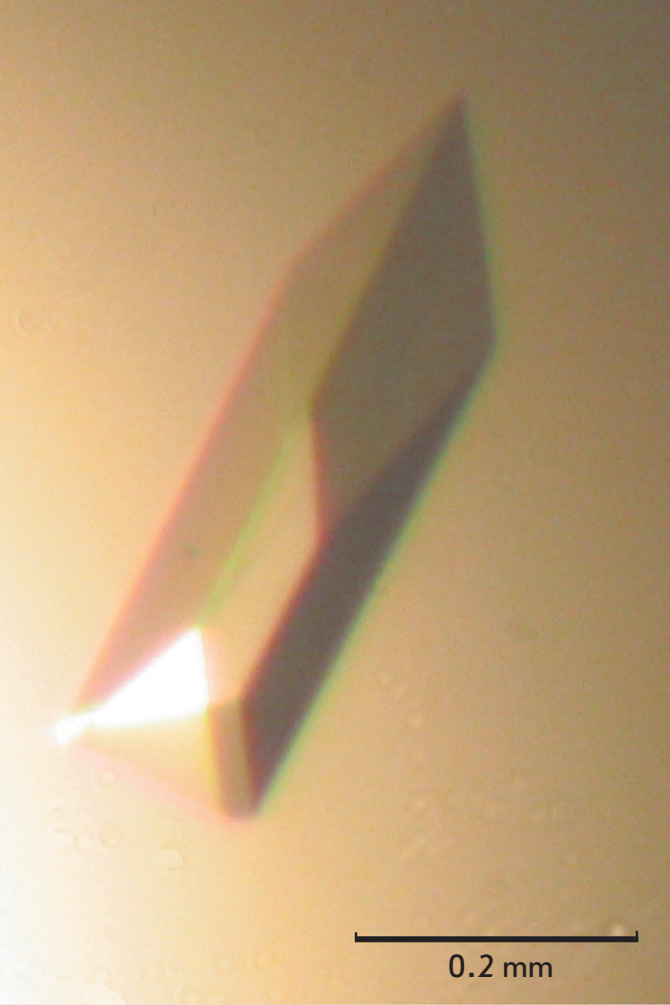
Photograph of crystals of the ternary MorFDH-NAD^+^-azide complex

Crystals of the MorFDH-NAD+-azide ternary complex were grown by the hanging drop vapor diffusion method. Recombinant full-size MorFDH was expressed and purified according to the procedure described in [14]. The purity of the enzyme estimated by polyacrylamide gel electrophoresis was at least 97%. The crystallization was performed using a solution of MorFDH at a concentration of 10.5 mg/ml in 0.1 M potassium phosphate buffer, pH 7.0, containing 5 mM NAD+ and 5 mM sodium azide. The reservoir solution consisted of 0.1 M Bis-Tris buffer, pH 6.5, and 2.0 M ammonium sulfate. The hanging drops (4 ≤l) were prepared by mixing equal volumes of the protein and reservoir solutions on siliconized glass cover slides. Wells of plastic Linbro plates (Hampton Research) were filled with 500 ≤l of the reservoir solution, and the cover slides were inverted and sealed over the wells. The plates were stored in a temperature-controlled cabinet at 20 °C. The crystals grew within two months to an average size of 0.6 x 0.3 x 0.2 mm (Fig. 1).

X-ray diffraction data were collected at a wavelength of 0.8166 A using a MAR165 CCD detector at the EMBL X11 beamline at the DORIS storage ring of the DESY synchrotron (Hamburg). To decrease radiation damage in the protein crystal and increase the resolution due to a decrease in the thermal displacements of the atoms, the crystal was cooled with nitrogen (100 K) using a Cryojet cryocooler during data collection. Before freezing in a stream of nitrogen, the crystal was soaked in a cryosolution (0.1 M Bis-Tris buffer, pH 6.5, 2.3 M ammonium sulfate, and 30 % (v/v) glycerol) for 30 s. Two X-ray diffraction data sets were collected from the same crystal. The first data set was collected in the resolution range 20-1.5 A with a short exposure time; the second data set, in the resolution range 20-1.1 A with a longer exposure time. This made it possible to avoid overexposure of low-resolution reflections, which is often the case when the exposure time is high, and to measure weak high-resolution reflections with high accuracy. The diffraction data sets were processed with DENZO and SCALEPACK program packages [15]. The crystals belong to the space group C2 with the unit-cell parameters a = 79.2 A, b = 66.2 A, c = 74.2 A, and ≤ = 103.4^o^.

Since these crystals are isomorphous with the crystals that were used for the structure determination of the MorFDH-NAD^+^-azide ternary complex at 1.95 A resolution [14], the latter structure was used as the starting model. The structure refinement was performed using the REFMAC program [16]. The contribution of hydrogen atoms to the X-ray scattering was included in the refinement. The coordinates of the hydrogen atoms were calculated based on the known stereochemistry of amino acid residues and the coordinates of the corresponding covalently bonded atoms in each refinement cycle. The temperature factors for all non-hydrogen atoms were refined anisotropically. The progress of the refinement was monitored, the atomic model of the structure was visually inspected, and water molecules were located using the COOT program [17]. The corrections were made based on the analysis of difference Fourier maps with the coefficients (2|Fo| - |Fc|) and (|Fo| - |Fc|, where |Fo| and |Fc| are the observed and calculated structure-factor amplitudes. The quality of the protein model was analyzed using the PROCHECK program [18]. The errors in the atomic coordinates were calculated using the SFCHECK program [19]. The diffraction data collection and atomic model statistics are summarized in Table 1.

## RESULTS AND DISCUSSION

**Fig. 2.**
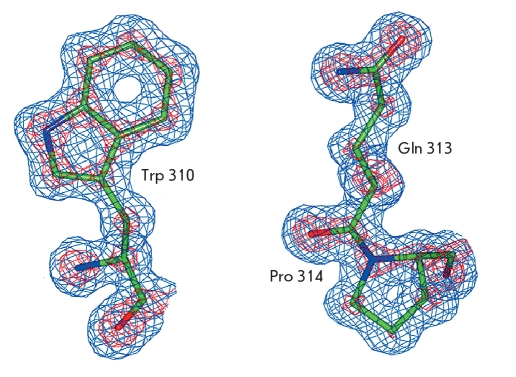
Electron density map with 2|Fo| - |Fc| coefficients for residues Trp310, Gln313, and Pro314. The map is contoured at 1.0≤ (blue) and 3.5≤ (red) levels

The great advantage of atomic resolution is the high ratio of observations to parameters, due to which the temperature factors of non-hydrogen atoms can be refined anisotropically. In the isotropic approximation, three positional parameters and the isotropic temperature factor of each non-hydrogen atom are refined, whereas six anisotropic thermal parameters are used instead of one isotropic temperature factor for each atom (three of these parameters provide the orientations of the principal axes of the ellipsoid; the other three parameters represent the magnitudes of displacement along the ellipsoid axes) in the anisotropic refinement. This approach allows a much more accurate interpretation of the diffraction data. The inclusion of the anisotropic parameters into the crystallographic refinement of the MorFDH-NAD^+^-azide structure lowered the R factor by 5.3 % and the Rfree, by 5.2 %. The mean weighted error of the atomic coordinates characterized by the diffraction precision index (DPI) also decreased from 0.036 to 0.028 A. For the previously published structure of the MorFDH-NAD+-azide complex at 1.95 A resolution, this value was 0.141 A [14]. The high quality of the electron density maps for the atomic resolution structure is demonstrated in Fig. 2.

**Fig. 3.**
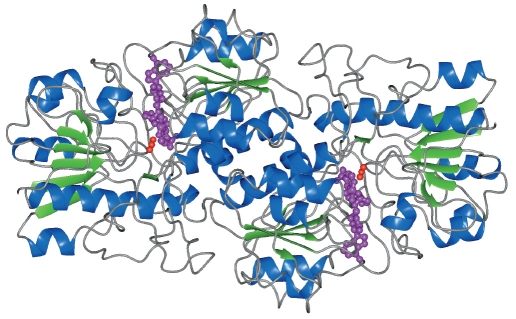
Ribbon representation of the the ternary MorFDH-NAD^+^-azide complex. The subunits of the MorFDH dimer are related to each other by the crystallographic twofold rotation axis perpendicular to the plane of the figure. The NAD^+^ molecule is depicted in purple, the azide ion is depicted in red

The polypeptide chain fold of the solved structure of the ternary MorFDH-NAD^+^-azide complex is depicted in Fig. 3. The asymmetric unit cell contains one subunit of the enzyme. The MorFDH molecule consists of two subunits related by a crystallographic twofold rotation axis (Fig. 3). In the crystal structure, 399 amino acid residues, 437 water molecules, one NAD^+^ molecule, four azide ions, and six glycerol molecules were located. The NAD ^+^ molecule and one azide ion are bound in the active site of the enzyme, whereas three azide ions and all glycerol molecules are located on the surface of the protein globule. Like in the structure solved at 1.95 A resolution, the C-terminal residues 392-399 were located in the atomic resolution structure. These eight residues were not visible in electron density maps calculated for the previously solved structure of the holo-form of FDH from the bacterium Pseudomonas sp. 101 [12]. Nevertheless, the last two residues were not located in the atomic resolution structure as well, which is probably due to the disorder of these residues.

The rms deviation of the coordinates of all C≤ atoms of the MorFDH-NAD^+^-azide structures solved at 1.1 A and 1.95 A resolution is 0.30 A, which indicates that they are identical. The maximum deviation is 1.1 A (for the residue Ser18). The atomic coordinates for 18 amino acid side chains located on the surface of the protein globule differ by more than 1 A, with the maximum deviation being 9 A (for the residue Arg26). These differences may be associated with the properties of the crystals grown in slightly different crystallization conditions or by the erroneous location of the side chains in the structure at lower resolution.

A considerable difference of the structure at atomic resolution is that the number of water molecules located in this structure is 2.4 times larger than that in the structure solved at 1.95 A resolution (437 and 181 molecules, respectively). It should be noted that 169 water molecules have virtually identical positions in both structures. In fact, only conserved highly ordered water molecules, which are located in the cavities of the protein globule and in the first solvent shell, were located in the structure at 1.95 A resolution. The atomic resolution structure allowed the much more accurate and reliable determination of the solvent structure in the crystal structure of the MorFDH-NAD+-azide complex. 

Due to the high accuracy of diffraction data, the atomic resolution allows the more precise determination of the alternative conformations of residues. In the structure of the MorFDH-NAD^+^-azide complex at 1.95 A resolution, only five residues were found to adopt two different conformations. A considerable improvement of the quality of the electron density maps for the atomic resolution structure allowed the identification of ten such residues. The side chains of five lysine residues (Lys40, Lys61, Lys231, Lys383, and Lys395) and one glutamine residue (Glu397) were located only partially, which is evidence that these residues exist in several conformations. All residues adopting alternative conformations and with missing side chain atoms in the model are on the surface of the protein globule, which accounts for their high conformational flexibility.

A considerable advantage of atomic resolution diffraction data is that carbon, oxygen, and nitrogen atoms can be reliably differentiated, which allows the identification of rotational isomers of asparagine, glutamine, and histidine side chains. The rotational isomers of these residues are usually determined by analyzing the hydrogen bonds with adjacent atoms, and in some case definite conclusions cannot be drawn. The X-ray diffraction data at atomic resolution allows one to determine the isomers of asparagine and glutamine residues by analyzing the temperature factors of the side chain nitrogen and oxygen atoms. If the carboxamide group of asparagine or glutamine in the model is rotated by 180^0^ with respect to the actual conformation in the protein crystal, the B factor of the nitrogen atom will be considerably lower, whereas the B factor of the oxygen atom will be considerably higher than the average temperature factors of covalently bonded atoms. A similar situation (higher B factors of the nitrogen atoms N≤ and N≤ and lower B factors of the carbon atoms C≤ and C≤) is observed also for incorrectly rotated histidine side chains. In addition, the diffraction data at atomic resolution allows one to differentiate between carbon, oxygen, and nitrogen atoms based on electron density maps, because atoms with more electrons have higher electron density peaks (Fig. 2). To check for rotational isomers of asparagines, glutamine, and histidine, the analysis of electron density maps was accompanied by a separate refinement cycle using a modified model, in which all side chains of these residues were rotated by 180°. The rotational isomers of most residues in the structure of the MorFDH-NAD^+^-azide complex at 1.1 A resolution were confirmed, and the side chains of the Gln66 and Asn135 residues were found to be rotated compared to their conformation in the 1.95 A resolution structure. It was not always possible to unambiguously choose the isomer based on the analysis of the temperature factors, which is an indication that both rotational isomers are present in the structure.

**Fig. 4.**
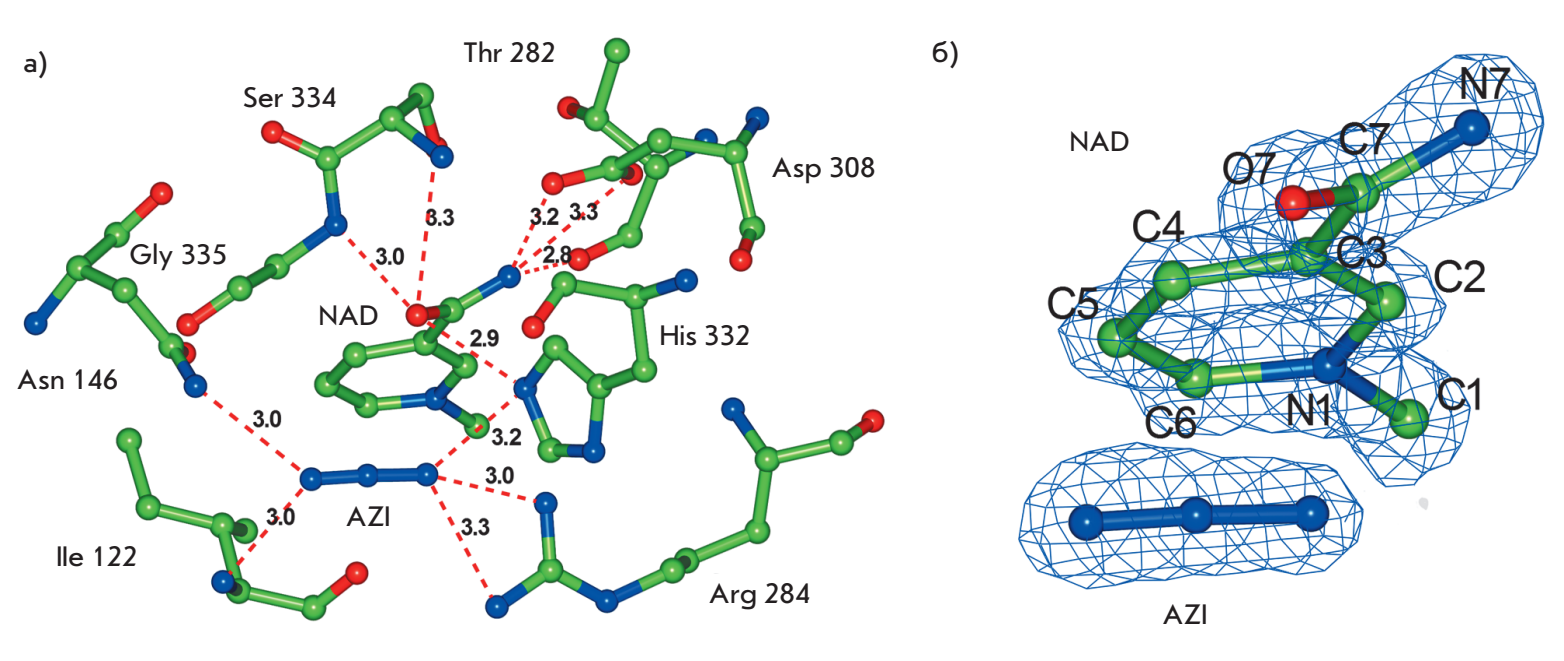
Structure of the active site of MorFDH with the bound NAD^+^ molecule and azide ion a) Binding of the nicotinamide moiety of the NAD^+^ molecule and the azide ion by the active site residues (hydrogen bonds are depicted as dotted lines). b) Electron density map with 2|F0| - |Fc| coefficients countered at the 2.0≤ level for NAD^+^ and azide and the atomic numbering scheme for the nicotinamide moiety of NAD ^+^

The structure of the active site of MorFDH containing the bound NAD^+^ molecule and azide ion is depicted in Fig. 4. A detailed description of the structure of the FDH active site and the role of individual amino acid residues in the binding of the substrates and the catalysis can be found in the studies [12, 14]. The kinetic isotope effects study showed that the hydride ion transfer is the rate-limiting step of the enzymatic reaction, the transition state being the late one i.e, it is structurally more similar to the reaction products [20, 21]. The linear azide ion is isoelectronic with the reaction product CO2, which is also linear. Moreover, azide ion is characterized by an extremely high binding constant to the holo-FDH, which is about five orders of magnitude higher than that of the formate ion [20]. Hence, the ternary MorFDH-NAD^+^-azide complex is considered as a stable analog of the transition state of the enzymatic reaction [20, 22].

The X-ray diffraction data at atomic resolution confirm that the carboxamide group of the cofactor in the active site of MorFDH is fixed in the trans conformation (the 07 atom is directed to the C4 atom) by hydrogen bonds with active site residues. According to quantum-mechanical calculations in the gas phase, the trans conformation is 2 kcal/mol less favorable than the cis conformation [23]. The analysis of the refined structure showed that the length of the C7-07 double bond in the carboxamide group of the cofactor is 1.26 A, which is 0.03 A longer than the standard C-0 double bond length in the carboxamide group (1.23 A) [24]. At the same time, the length of the C3-C7 single bond is 1.47 A, which is 0.03 A smaller than the standard value (1.50 A). Since the rms deviation for covalent bonds in the structure is 0.015 A, these changes in the bond lengths between the atoms with relatively low temperature factors (07 - 15.6 A2, C7 - 13.7 A2, C3 - 14.1 A2) inside the protein molecule may be an indication of a change in the bond orders. These previously unknown characteristics of the MorFDH-NAD^+^-azide ternary complex may reflect important details of the structural organization of the transition state in the FDH-catalyzed reaction.

Previously, it has been shown by calculation methods, using combined molecular dynamics and quantum mechanics studies, that the cofactor molecule adopting the energetically activated trans conformation in the transition state of the FDH-catalyzed reaction may take on the properties of the so-called bipolar form [4]. A similar hypothesis was also suggested on the basis of the kinetic isotope effects analysis [21]. The bipolar form is characterized by a higher negative charge on the O7 atom of the carboxamide group, the shorter C3-C7 bond, and the longer C7-07 bond compared to those in the free cofactor molecule. These changes promote the increase in the partial positive charge on the C4 atom of the coenzyme, thus increasing its electrophilicity and facilitating the rate-limiting hydride ion transfer and the enzymatic reaction. 

Due to the high accuracy of the atomic resolution diffraction data, the influence of standard stereochemistry on the atomic coordinates of the nicotinamide group of the cofactor can be weakened in the course of the crystallographic structure refinement. After a separate refinement cycle using the REFMAC program with weaker bond length constraints for the nicotinamide group (by increasing the rms bond length deviation from 0.02 A to 0.20 A), the C7-07 bond length was 1.29 A, which is 0.06 A smaller than the standard value, and the C3-C7 bond length was 1.43 A, which is 0.07 A longer than the standard value. These differences are only 1.3 times larger than the error in the bond lengths estimated as the sum of two mean-weighted errors in the atomic coordinates (Table 1). Nevertheless, our experimental data reveal a tendency toward a change in the covalent bond lengths, which corresponds to the fact that the nicotinamide group of the cofactor adopts the bipolar form in the transition state of the enzymatic reaction.

**Table 1:** X-ray diffraction data and atomic model statistics. Values in parentheses are for the highest-resolution shell (1.11 - 1.10 A).

Resolution, Aring;	20 - 1.1
No. of observed reflections	586345 (9155)
No. of unique reflections	145564 (3662)
Mosaicity, Aring;	0.7
Redundancy	4.0 (2.5)
Completeness, %	96.5 (73.8)
R_merge_, %	5.1 (52.9)
<I>/<σ(I)>	44 (1.9)
B factor from Wilson plot, Aring;^2^	11.5
R_work_/R_free_, %	13.4/15.9
R.m.s. deviation of covalent bonds from ideal, Aring;	0.015
R.m.s. deviation of covalent angle from ideal, Aring;	1.7
Minimal coordinate error, Aring;	0.014
Diffraction precision index (DPI), Aring;	0.028
No. of non-H atoms: protein water ligands	3106 437 84
Average B factors, A^2^: all atoms main-chain atoms side-chain atoms water molecules ligand atoms	20.7 18.1 20.1 31.8 24.9
No. of residues in Ramachandran plot^*^: in most favored regions in additional allowed regions in disallowed regions	306 33 1^**^

* Except for Gly and Pro residues ** Ala198 in the FDH structure is outside the allowed region. The unusual conformation of this residue is discussed in detail in [12].

To sum up, the atomic resolution structure of FDH was determined for the first time, which provides a deeper insight into the details of the molecular mechanism of this enzyme and the hydride ion transfer in the active sites of NAD^+^-dependent dehydrogenases.

## Acknowledgitalicents

This work was supported by the Russian Federal Agency for Science and Innovation (federal contract 02.512.12.2002) and the Russian Foundation for Basic Research (grant № 08-04-00830-a). The authors thank the italicBL Hamburg Outstation for the allotted time on the synchrotron radiation source and Alexander Popov personally for assistance with diffraction data collection.
